# Design and comparative analysis of laser-based array systems for UAV detection in surveillance zones

**DOI:** 10.1371/journal.pone.0325752

**Published:** 2025-06-25

**Authors:** Meriem Salhi, Maha Sliti, Noureddine Boudriga, Abdelrahman Elfikky, Sarra Ayouni

**Affiliations:** 1 University of Carthage, Higher School of Communication of Tunis (SUP’COM), LR11TIC04, Communication Networks and Security Research Lab. & LR11TIC02, Green and Smart Communication Systems Research Lab, Ariana, Tunisia; 2 University of Carthage, Higher School of Communication of Tunis (SUP’COM), Ariana, Tunisia; 3 Arab Academy for Science and Technology and Maritime Support, Alexandria, Egypt; 4 Department of Information Systems, College of Computer and Information Sciences, Princess Nourah bint Abdulrahman University, P.O. Box 84428, Riyadh, 11671, Saudi Arabia; inSync Mirror LLC, UNITED STATES OF AMERICA

## Abstract

Identifying unmanned aerial vehicles (UAVs) is critical to protecting vital locations and infrastructures from potential attacks. The literature suggests a variety of detection methods, including conventional radar systems, acoustic detection, radio frequency signal detection, LiDAR, and camera-based techniques. LiDAR systems, in particular, offer high-resolution 3D mapping and precise distance measurements, which prove to be highly effective for detecting and tracking UAVs under various environmental conditions. This study presents two innovative LiDAR systems for UAV detection: a multi-array static LiDAR system and a one-array rotating LiDAR system. The multi-array static LiDAR employs arrays of laser light sources and concentrators arranged along a spherical shape. A central photodiode receives the transmittance of the reflected optical energy captured by the concentrators, enabling the precise identification of UAVs. The system’s design focuses on achieving continuous, high-resolution coverage with minimal delay, making it suitable for monitoring wide areas. In contrast, the one-array rotating LiDAR utilizes a single array with rotational motion to scan the surveillance area. This approach prioritizes compactness and energy efficiency, which makes it advantageous for cost-sensitive applications. However, the rotational mechanism introduces trade-offs, such as increased mechanical wear and scanning latency. By conducting a comprehensive analysis of the design characteristics, this study evaluates the practicability and efficiency of these LiDAR solutions. Parameters such as the dimensions of the monitored region, sensor characteristics, component arrangement, and interspacing are considered to assess the effectiveness of both systems in identifying potential UAV threats.

## 1 Introduction

In recent years, the proliferation of unmanned aerial vehicles (UAVs), commonly known as drones, has led to an increased need for advanced surveillance and detection systems. Drones, while offering numerous benefits in fields such as transportation, delivery services, and emergency response, also present significant challenges to security, especially in sensitive areas such as airports, military bases, and critical infrastructure [1, 2]. The threat posed by unauthorized or malicious UAVs, which can evade traditional detection methods, has required the development of more sophisticated and reliable systems to detect, track and potentially neutralize these airborne threats [3].

Traditional drone detection methods, such as radar, acoustic sensors, and radio frequency (RF) systems, have been widely used. However, these techniques often face limitations in terms of range, accuracy, and susceptibility to environmental interference [4]. For example, radar systems can struggle to differentiate between UAVs and other objects in the air, such as birds or weather phenomena. Acoustic sensors may suffer from high noise levels in urban areas, whereas RF systems are dependent on signal strength and may be ineffective when drones operate autonomously or in environments with minimal communication signals [5]. Consequently, there has been growing interest in alternative detection technologies that can offer improved precision and reliability [6].

One promising solution is the use of laser-based detection systems, particularly those employing multiarray configurations. Laser systems offer several advantages over traditional techniques [7–10], including high spatial resolution, rapid data acquisition, and the ability to operate effectively in both day and night conditions [11]. These systems work by detecting optical light that is reflected off a flying target, allowing accurate distance measurements, tracking of movement, and classification of the object [12, 13]. The key advantage of laser-based detection lies in its precision and its ability to detect smaller objects at longer ranges compared to other sensors, which makes it highly suitable for the surveillance of airborne targets such as drones [14].

Despite the potential of laser-based systems, their performance is highly dependent on system design, dimensioning, and configuration. The choice of laser array type, the number of sensors, the alignment and coverage of the detection zone, and the system’s ability to process the reflected light data are all critical factors that determine the system’s effectiveness [15]. Furthermore, these systems must be designed to operate efficiently in real-world environments, where challenges such as varying target sizes, speeds, altitudes, and environmental conditions (e.g., weather, lighting) must be taken into account.

In this work, we extend our work proposed in [16, 17]. Thus, we propose a laser-based radar system, which is constructed of laser-emitting transmitters and laser optical concentrators connected to a photodiode. The main purpose of the proposed system is to detect, at least in the shortest possible time, a flying object that may potentially be an UAV. This paper presents two alternative laser-based array systems designed to detect the optical light reflected from a flying target within a defined surveillance zone. The first system utilizes a fixed laser array with a wide field of view, while the second system incorporates a dynamic array configuration that adjusts based on the detected target’s position. Both systems are analyzed in terms of their design specifications, operational principles, and detection capabilities. We explore the system dimensioning process in detail, highlighting key parameters that influence the detection performance, including laser intensity, sensor placement, and array size. By understanding how these factors interact, we aim to identify the optimal configuration to maximize detection efficiency. In addition, this paper conducts a comparative analysis between the two proposed approaches, evaluating their respective benefits, drawbacks, and performance in various scenarios. The impact of dimensioning factors on detection efficiency is also discussed, providing a comprehensive overview of the trade-offs involved in selecting the best system for different use cases. Through this analysis, our aim is to offer valuable insights into the design and implementation of laser-based detection systems, contributing to the growing body of research in the field of UAV surveillance.

The remainder of the paper is organized as follows. Sect 2 presents recent advances in laser-based systems related to flying target detection. Sect 3 presents the considered system model. Sects 4 and 5 present, respectively, the turning Lidar alternative and the static Lidar alternative. We provide a comparison overview of the two alternatives in Sect 6. The obtained simulation results are presented and discussed in Sect 7. Finally, Sect 8 concludes the paper.

## 2 Related work

The increasing prevalence of UAV-related security threats, including unauthorized surveillance, smuggling, and potential attacks on critical infrastructure, has highlighted the urgent need for robust detection and mitigation systems. These threats pose significant risks to public safety, national security, and restricted airspaces, emphasizing the need to advance UAV detection technologies to ensure comprehensive surveillance and protection measures. Recent studies [12, 18–21] have reviewed UAV-related security incidents and defense strategies, underscoring the critical demand for advanced detection frameworks to counteract malicious UAV operations.

In response to these concerns, recent advances in laser-based detection systems have significantly enhanced UAV tracking capabilities in surveillance zones. Multiarray architectures, such as the spherical LiDAR systems proposed by Salhi and Boudriga [16], have demonstrated improved spatial coverage and detection precision by capturing data from multiple angles. These systems facilitate robust tracking of dynamic targets in complex airspaces, addressing the limitations of single-sensor configurations. Similarly, Kim and Jang [22] introduced a LiDAR-based drone detection system, serving as a foundational framework to optimize UAV identification techniques. The integration of 360° LiDAR systems for counter-UAV missions, as explored by Paschalidis *et al*. [23], further underscores the adaptability of laser-based technologies in real-time aerial surveillance.

One of the primary factors influencing LiDAR-based UAV detection is the performance of laser radar systems in terms of range accuracy and echo characterization. Xu *et al*. [24] examined the role of beam coherence and environmental factors in determining laser measurement precision, while Dogru and Marques [25] highlighted the efficiency of sparse LiDAR data in reducing computational overhead without sacrificing detection accuracy. Furthermore, the application of LiDAR enhancements for specialized challenges has been explored through techniques such as removing swarm noise from drone-acquired data using PointPillars, as demonstrated by Dow *et al*. [26]. Hardware innovations have also contributed to improved LiDAR performance, with advanced photodetectors that improve detection sensitivity and adaptability to varying environmental conditions [27].

Environmental factors present a major challenge in UAV detection, requiring LiDAR systems to maintain accuracy under adverse weather conditions. Du *et al*. [28] investigated the performance of LiDAR in rainy and foggy conditions, demonstrating the reliability of the system in reduced visibility. Similarly, Wang *et al*. [29] explored the integration of tracking methods with LiDAR, offering a holistic approach to UAV detection by improving resilience in real-world environments. Lian *et al*. [30] extended this research by assessing the functionality of LiDAR in dusty weather, reinforcing the need to optimize LiDAR signal processing techniques for enhanced stability in complex atmospheric conditions. Recent advances in SAR signal processing, such as the range ambiguity suppression method using blind source separation (BSS) proposed by [31], and the advanced echo separation scheme for space-time waveform-encoding SAR with digital beamforming [31], can significantly improve UAV detection in complex environments. These techniques help mitigate interference and reduce signal distortion, improving data clarity and the robustness of SAR systems for accurate UAV tracking, particularly in urban or cluttered settings. In UAV-based optical wireless communication, environmental factors such as atmospheric turbulence, pointing errors, and scintillation effects significantly impact system performance [32, 33]. Recent research by Hayal *et al*. has analyzed how these factors influence hovering UAV-based FSO relay systems, providing insights into performance improvement strategies under real-world turbulence conditions [34]. Similarly, Elsayed investigated OFDM-based FSO systems for UAV-to-ground communication, focusing on scintillation mitigation techniques to maintain signal integrity in turbulent environments [35–37]. These studies highlight the critical role of environmental effects in UAV-based optical sensing and communication, reinforcing the need for robust LiDAR-based UAV detection models that account for similar challenges in practical deployments.

Beyond LiDAR, complementary sensor technologies, such as acoustic and radar-based systems, offer valuable integration opportunities to improve UAV detection accuracy [36–46]. For example, Hammer *et al*. [47] highlighted the benefits of multi-sensor fusion, combining LiDAR with acoustic arrays for enhanced drone localization and improved tracking performance in cluttered environments. Similarly, recent research has demonstrated the effectiveness of fusion-based models such as TransFusion and BEVFusion, which leverage cross-sensor data fusion to produce more reliable UAV tracking in complex operational settings [48–50].

The application of machine learning in UAV detection has led to substantial improvements in LiDAR-based classification and tracking algorithms. Mrabet *et al*. [12, 51] demonstrated how deep learning techniques enhance real-time UAV detection and classification, enabling more accurate discrimination between UAVs and non-threatening airborne objects. Ojdanić *et al*. [52] further explored the integration of camera-guided laser ranging, combining visual and laser-based data to improve multi-UAV distance measurement. These developments, along with the use of physical simulation models for adverse environmental conditions [30], highlight the robustness of deep learning-enhanced LiDAR detection methods. Similarly, Su *et al*. [53] applied convolutional neural networks (CNNs) to LiDAR point clouds, achieving high detection accuracy and operational reliability. Huang *et al*. [54] provided a comprehensive review of LiDAR-based target detection methods using deep learning, positioning specific studies within the broader landscape of AI-driven LiDAR advancements.

Emerging innovations in LiDAR hardware and system design are further improving UAV detection performance. Khuseynzada *et al*. [27] explored the development of novel photodetectors, which optimize signal acquisition and processing for next-generation LiDAR systems. Pritzl *et al*. [55] investigated cooperative navigation using 3D LiDAR, demonstrating its potential for multi-agent UAV detection. Furthermore, MEMS-based LiDAR systems, such as the synchronized dual-laser beam system proposed by Huang *et al*. [56], have significantly extended detection range and angular coverage. Advancements in laser diode array driver circuits, such as those presented by Li *et al*. [57], further contribute to improving LiDAR efficiency and real-time tracking capabilities.

Recent advances in SAR signal processing have demonstrated innovative approaches to managing signal interference and distortion. For example, Chang *et al*. [31] introduced an advanced scheme for suppression of range ambiguity of spaceborne SAR based on blind source separation, which effectively isolates overlapping echoes to improve range resolution. Similarly, Chang *et al*. [58] developed an advanced echo separation scheme for space-time waveform encoding SAR that leverages digital beamforming combined with blind source separation, offering improved robustness against interference and signal distortion. These studies provide valuable insights into the latest techniques for managing echo overlap and range ambiguity. Their approaches could be adapted to further enhance the robustness of our LiDAR-based UAV detection system, particularly in environments with multiple interference sources. Future work may explore the integration of these advanced signal processing methods to mitigate distortions and improve overall detection performance.

Despite these advancements, several limitations persist in current LiDAR-based UAV detection approaches. Existing research predominantly focuses on either static or rotating LiDAR systems without a direct comparative analysis of their respective advantages and trade-offs. In addition, few studies incorporate detailed environmental modeling, such as the impact of atmospheric turbulence, beam divergence effects, and real-time signal degradation. Furthermore, most existing approaches fail to simultaneously evaluate key performance metrics, including detection efficiency, power received, and coverage range, making it difficult to determine the optimal LiDAR architecture for UAV surveillance applications. Table 1 gives a comparative Overview of LiDAR-Based UAV Detection Technologies and Approaches.

**Table 1:** Comparative overview of LiDAR-based UAV detection technologies and approaches.

Aspect	Study	Key Insights	Challenges Addressed	Future Potential
LiDAR-Based Detection	Salhi and Boudriga [16]	Spherical LiDAR systems for enhanced spatial coverage and detection precision.	Improved spatial coverage and precision in complex airspaces.	Advanced spherical LiDAR for dynamic target tracking.
	Kim and Jang [22]	Framework for UAV identification using LiDAR.	Foundational steps for optimized detection.	Application of LiDAR for broader surveillance scenarios.
	Paschalidis *et al*. [23]	360° LiDAR for counter-UAV missions.	Robust detection in modern surveillance applications.	Integration into military and critical infrastructure defenses.
Innovative Hardware	Khuseynzada *et al*. [27]	Development of innovative photodetectors for LiDAR systems.	Improved detection sensitivity and environmental adaptability.	Photodetectors tailored for specific UAV detection challenges.
	Pritzl *et al*. [55]	Cooperative navigation using 3D LiDAR for micro-scale aerial vehicles.	Collaboration in UAV management and detection.	Enabling multi-UAV systems and cooperative tracking.
	Huang *et al*. [56]	MEMS-based LiDAR system with synchronized dual laser beams.	Improved detection range and accuracy.	Enhanced LiDAR systems for long-range and high-precision applications.
	Li *et al*. [56]	Nanosecond pulse laser diode array driver circuit for LiDAR systems.	Enhanced LiDAR efficiency and performance.	LiDAR systems with optimized power usage and faster response.
Machine Learning Advances	Mrabet *et al*. [12]	Machine learning for real-time UAV detection and classification.	Discrimination between UAVs and other aerial objects.	Real-time adaptability with advanced AI models.
	Su *et al*. [53]	CNNs applied to LiDAR point clouds for high detection accuracy.	Improved classification and reliability in diverse conditions.	Deployment in large-scale UAV surveillance systems.
Environmental Resilience	Du *et al*. [28]	Performance of LiDAR systems in rainy and foggy conditions.	Reliability in adverse weather conditions.	Robust UAV detection under extreme environmental conditions.
	Wang *et al*. [29]	Integration of tracking methods with LiDAR systems.	Holistic approach to UAV monitoring.	Development of comprehensive surveillance frameworks.
	Dow *et al*. [26]	Removal of swarm noise using PointPillars algorithm.	Improved signal clarity in drone-acquired data.	Enhanced machine learning models for noise filtering.
	Xu *et al*. [24]	Impact of beam coherence and environmental factors on laser measurement precision.	Range accuracy and echo characterization improvements.	Development of adaptive LiDAR systems for diverse conditions.
	Dogru and Marques [25]	Sparse LiDAR data for reduced computational overhead.	High efficiency with lower resource requirements.	Expanded applications in low-resource environments.
Multi-Sensor Integration	Hammer *et al*. [47]	Benefits of combining LiDAR with acoustic arrays.	Improved localization and tracking in complex conditions.	Synergistic use of acoustic and LiDAR data for UAV tracking.
	Chang *et al*. [50]	Acoustic arrays for drone localization.	Enhanced accuracy in challenging environments.	Integration with other modalities for holistic UAV monitoring.

To address these gaps, our study presents the first direct comparative analysis of static and rotating LiDAR systems for UAV detection. Unlike previous studies that examined these configurations separately, our work quantifies their relative performance differences in terms of detection efficiency, power reception, and response time. By optimizing sensor placement, rotation speed, and inter-array spacing, our study provides practical deployment strategies for LiDAR-based UAV detection. Furthermore, by comparing instantaneous detection (static LiDAR) vs. sequential scanning (rotating LiDAR), we establish clear guidelines for selecting the best system configuration based on surveillance area, detection latency, and operational constraints. The following sections detail our system model and methodology, outlining the key design considerations for both LiDAR configurations.

## 3 System model

In developing our LiDAR-based UAV detection algorithm, several key assumptions were made to simplify the system design and facilitate comparative analysis. First, for the noise model, we assume an atmospheric transmittance of 0.8 and a UAV surface reflectivity of 0.9, which reflect moderate environmental conditions (such as haze, light fog, or dust) commonly encountered in urban and semi-urban environments. The use of a 1550 nm laser wavelength was chosen to minimize atmospheric absorption while ensuring eye safety, thus making the system suitable for surveillance applications. Second, regarding system geometry, our design employs a quasi-spherical arrangement of laser sources and optical concentrators to achieve 360° azimuthal coverage and wide elevation range. This configuration is further optimized by explicitly considering beam divergence trade-offs: a narrow beam improves detection range due to higher energy concentration, while a wider beam increases coverage at the cost of signal intensity. Sensor placement, particularly the inter-array spacing in the multi-array static LiDAR, is optimized to balance sensor overlap with coverage continuity, with an optimal inter-array angle range identified between 180° and 240°. Third, in modeling target dynamics, it is assumed that UAVs enter the surveillance zone randomly, moving along linear trajectories at a constant speed (with 15 m/s serving as a representative value for small to medium UAVs). For the one-array rotating LiDAR system, the impact of the rotation period on detection latency is explicitly addressed; for instance, increasing the turn duration beyond 7 seconds markedly reduces detection efficiency due to delayed coverage. Future work will focus on incorporating adaptive noise filtering techniques and real-time UAV motion prediction models to enhance robustness and account for more complex environmental and dynamic scenarios.

### 3.1 General system overview

**Fig 1:**
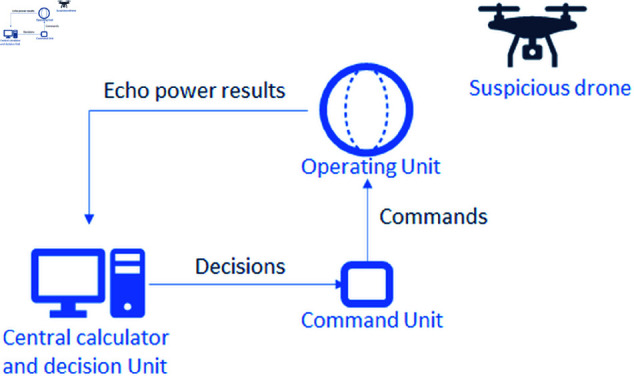
General system overview.

The proposed system consists of three main parts, as represented in Fig 1:

- **The Operating Unit:** This unit comprises a collection of laser photodiodes and receivers arranged on the surface of a quasi-spherical structure. Each photodiode is responsible for detecting the accumulation of optical echo powers.
- **The Command Unit:** This unit manages the operations of the operating unit by issuing control commands. These commands include the activation or deactivation of laser pulses, as well as the selection of emitting diodes or arrays.
- **The Central Calculator and Decision Unit:** This unit collects the optical echo power results from the operating unit, performs the necessary calculations, and makes decisions, which are then sent back to the command unit for execution.

### 3.2 Proposed architecture

The proposed system, represented in Fig 1, uses a quasispherical radar configuration, a design choice that offers significant advantages for UAV detection and tracking. One of the primary benefits of this architecture is its ability to provide comprehensive coverage. The quasi-spherical geometry enables a uniform spatial distribution of laser sources (emitters) and photodiodes with optical concentrators (sensors) across the structure. This ensures a 360 degree field of view in the azimuthal plane and extensive coverage in elevation, allowing the system to monitor targets effectively across a wide range of directions. Such omnidirectional detection is critical for surveillance applications, where UAVs may approach from unpredictable trajectories.

Another key advantage of the quasi-spherical design is its inherent redundancy. Multiple sensors positioned on the curved surface, specifically photodiodes with optical concentrators, observe the same target from different angles, significantly reducing the likelihood of blind spots or missed detections. This overlapping field of view not only enhances the reliability of the system but also ensures that critical information, such as target position and motion characteristics, is captured with greater accuracy. Redundancy also improves resilience, allowing the system to maintain functionality even if individual sensors or emitters experience temporary malfunctions.

In addition to its coverage and robustness, the quasi-spherical radar configuration is highly compact. By integrating the detection and emission components onto a single curved surface, the system minimizes its overall volume while maintaining high detection performance. This compactness makes it particularly suitable for deployment in environments where space is limited, such as urban areas, mobile platforms, or field operations. The efficient use of space, combined with the system’s comprehensive functionality, underscores the practicality and adaptability of this architectural choice.

The radar is characterized by a height *h* and a radius *r*. Along the vertical arc’s longitudinal line, one or more transmit-receive laser arrays are arranged. Each array includes laser emitting sources (transmitters) and optical concentrators (receivers) [59]. At the center of the structure is a photodiode that collects the optical powers relayed by the concentrators through an optical medium.

**Fig 2:**
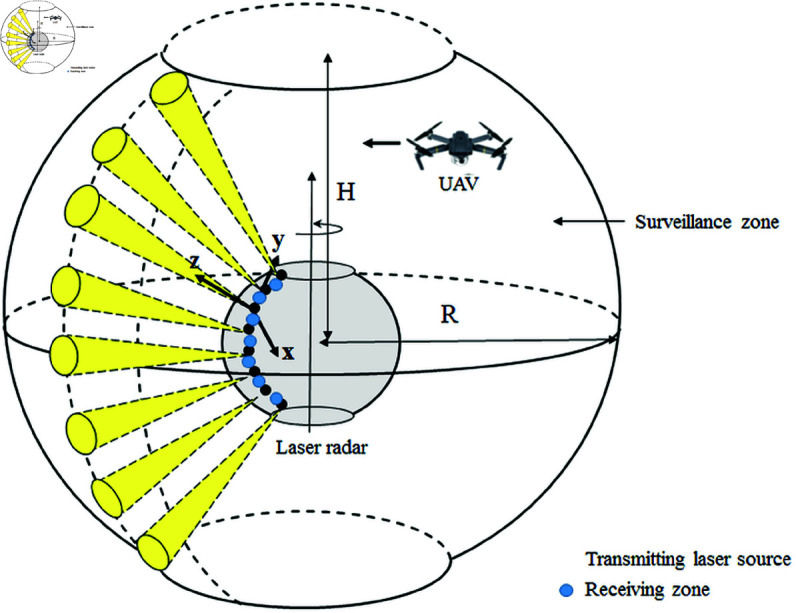
Proposed system radar architecture.

The considered observation zone has a radius *R* and a height *H*. Fig 2 illustrates the overall system architecture and its components. For the successful detection of an UAV, three conditions must be fulfilled:

1. The UAV must enter an illuminated region within the surveillance zone.
2. The reflected optical signal must reach one or more receivers.
3. The received optical power must exceed the detection threshold.

**Fig 3:**
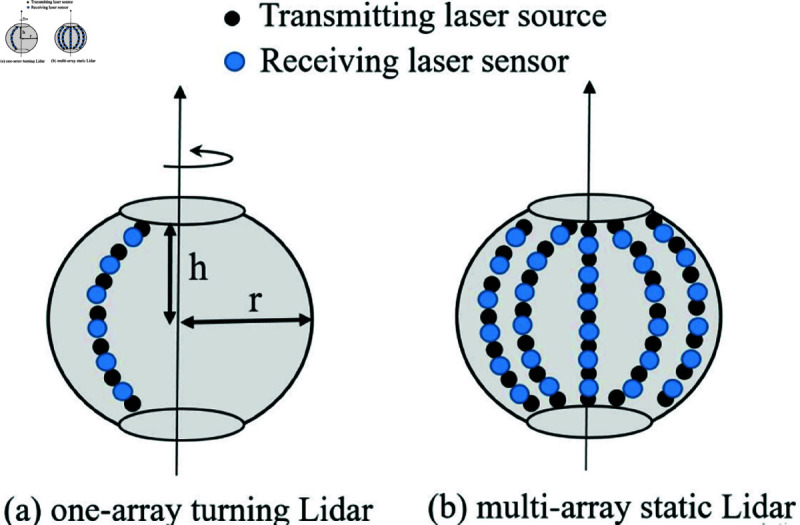
The LiDAR system alternatives.

Two design alternatives are considered for the lidar system, each with different operational characteristics and configurations. Fig 3 illustrates these alternatives:

- **The One-Array Turning Lidar Alternative:** This configuration consists of a single array of laser transmitters and receivers arranged on a spherical or quasi-spherical structure. To compensate for having only one array, the system incorporates rotational movement. This rotation allows the lidar beam to sweep through the surveillance area, effectively increasing the likelihood of detecting unmanned aerial vehicles (UAVs). The rotational mechanism improves coverage and detection efficiency without requiring additional arrays. However, the system may face challenges in terms of detection delay, as the rotation could introduce time gaps before revisiting specific regions in the surveillance zone.
- **The Multi-Array Static Lidar Alternative:** In this configuration, the lidar system is fixed and does not rely on any rotational movement. Instead, multiple arrays of laser transmitters and receivers are implemented to ensure comprehensive coverage of the surveillance area. The use of multiple arrays significantly increases the chances of detecting UAVs as each array operates independently to monitor a designated portion of the surveillance zone. This alternative is particularly advantageous in scenarios where instantaneous detection is critical. However, it may require a larger physical footprint and higher power consumption compared to the single-array turning lidar system.

### 3.3 Optical power model

**Fig 4:**
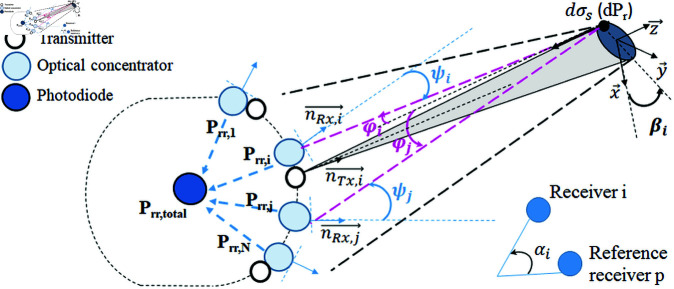
Transmit-receive system.

The transmit-receive system is illustrated in Fig 4. For each laser source, we define a local coordinate system where the *z*-axis aligns with the beam’s transmission direction. The target is modeled as an extended plane surface (i.e., a surface larger than the laser beam cross-section) [24]. Let *P*_*t*_ be the average transmitted power during a specific time interval.

#### 3.3.1 Reflected optical power and beam propagation.

A transmitting laser source *i* is located at a distance *R*_*t*_ from the reflective surface. Assuming the laser pulse is Gaussian in space and time, the transmitted power at time *t* over the pulse duration is expressed in Eq 1:

$$P_{t}(t)=P_{0}\exp\left(-\frac{t^{2}}{\tau^{2}}\right),$$ (1)

where τ is the pulse width.

The power intensity is distributed spatially according to the Gaussian distribution E(x,y,z) at a point (x,y,z), and the elemental optical power reflected from an area $d\sigma_{s}$ is expressed by Eq 2:

$$dP_{r}=E(x,y,z)P_{t}(t^{\prime })\eta_{atm}d\sigma_{s},$$ (2)

where $t^{\prime }=t-\frac{2R_{r}}{c}-\frac{2z}{c}$, and $\eta_{atm}$ is the atmospheric transmittance.

The Gaussian power intensity distribution is expressed by Eq 3:

$$E(x,y,z)=\frac{2P_{t}}{\pi \omega^{2}}\exp\left(-2\frac{x^{2}+y^{2}}{\omega^{2}}\right),$$ (3)

where ω is the beam radius at distance *z* given by Eq 4:

$$\omega =\frac{2\lambda }{\pi \phi_{0}}\sqrt{1+\frac{\pi \lambda z\phi_{0}^{2}}{4\lambda^{2}}},$$ (4)

with λ as the wavelength and $\phi_{0}$ as the beam divergence angle.

Assuming the reflecting surface is Lambertian, the laser cross-section of an elemental unit area is expressed by Eq 5:

$$d\sigma_{s}=4\rho \cos^{2}(\beta_{i})dS,$$ (5)

where ρ is the reflectance coefficient, $\beta_{i}$ is the angle of incidence, and *dS* is the surface area differential.

The spatial distribution of the laser beam is modeled using a Gaussian power intensity distribution (Eq 6):

$$E(x,y,z)=\frac{2P_{t}}{\pi \omega^{2}}\exp\left(-\frac{2(x^{2}+y^{2})}{\omega^{2}}\right)$$ (6)

where the beam radius, ω, is defined by Eq 7:

$$\omega =\frac{2\lambda }{\pi \phi_{0}}\sqrt{1+\frac{\pi \lambda z\phi_{0}^{2}}{4\lambda^{2}}},$$ (7)

with $\phi_{0}$ representing the beam divergence angle and *z* the distance from the source.

#### 3.3.2 Received echo power.

For a receiver *i* located at distance *R*_*r*_ from the reflecting surface, the channel’s direct current (DC) optical gain is expressed by Eq 8:

$$H_{i}(0)=\frac{(m+1)A_{PD}}{2\pi R_{r}^{2}}{\left(\cos\phi_{i}\right)}^{m}T_{s}g_{c}\eta_{atm}\cos\psi_{i}I_{\Psi }(\psi_{i}),$$ (8)

where *T*_*s*_ is the optical filter gain, *A*_*PD*_ is the photodiode surface area, *m* is the Lambertian order, and $I_{\Psi }(\psi_{i})$ is expressed by Eq 9:

$$I_{\Psi }(\psi_{i})=\left\{ \begin{array}{cc} 1, & \text{if }|\psi_{i}|\leq \Psi ,\\ 0, & \text{if }|\psi_{i}|>\Psi . \end{array}\right.$$ (9)

Here, Ψ is the concentrator’s field of view.

The elemental power received by receiver *i* from a reflecting surface *dS* is expressed by Eq 10:

$$dP_{rr,i}=H_{i}(0)dP_{r}.$$ (10)

The total received power is obtained by integrating the reflected power over the surface (Eq 11):

$$P_{rr,i}=4H_{i}(0)\rho \eta_{atm}\cos(\beta_{i})\int \int E(x,y,R_{r})P_{t}(t^{\prime })dxdy.$$ (11)

For an extended reflecting surface, following [24], the double integral simplifies to (Eq 12):

$$\frac{1}{2\omega \sqrt{2a}}\exp\left(-\frac{\xi_{1}^{2}}{c^{2}\tau^{2}}+\frac{b^{2}}{4a}\right),$$ (12)

where $\xi_{1}=2R_{r}-ct$ and the parameters *a* and *b* are expressed by Eqs 13 and 14:

$$a=\frac{2c^{2}\tau^{2}+4\omega^{2}\tan^{2}\beta_{i}}{c^{2}\tau^{2}\omega^{2}},$$ (13)

$$b=\frac{4\xi_{1}\tan\beta_{i}}{c^{2}\tau^{2}}.$$ (14)

## 4 First alternative: One-array turning lidar

This alternative involves the rotation of a single array of laser sources that transmit and receive laser signals in a 360-degree pattern along the vertical axis. This rotation enables the surveillance zone to be scanned by systematically illuminating various regions in all directions. Detection occurs when the illuminated zone intersects the surface of the flying UAV, and the UAV’s surface reflects a detectable amount of energy. The rotation of the array allows the lidar system to cover the entire surrounding environment, and each part of the zone is scanned as the array moves.

The detection efficiency depends on several factors, including the UAV’s mobility, the radar’s dimensions, the rotation velocity of the array, and the transmission characteristics of the laser, such as the beam divergence angle. These factors influence the ability to detect and track the UAV effectively over time. The configuration of rotating a single array reduces manufacturing costs and radar transmission energy by minimizing the number of laser sources and receivers. However, it requires additional power for the rotational kinetic energy.

### 4.1 Received echo power vector

The reflected optical power exhibits a Lambertian distribution, which depends on the beam width. Each concentrator *i* within the field of view (FOV) of the reflected beam, and at an angle $\psi_{i}$ within its FOV, receives a specific amount of power. Let $V_{P}$ denote the vector of received optical powers given by Eq 15:

$$V_{P}=(P_{rr,1},P_{rr,2},\ldots ,P_{rr,N})$$ (15)

where *P*_*rr*,*i*_ represents the optical power received at concentrator *i*. The total received power $P_{\text{total}}$ is given by Eq 16:

$$P_{\text{total}}=\sum_{i=1}^{N}P_{rr,i}$$ (16)

Detection is successful if $P_{\text{total}}$ exceeds a specified detection threshold $P_{\text{th}}$ given by Eq 17:

$$P_{\text{total}}>P_{\text{th}}$$ (17)

where $P_{\text{th}}$ represents the minimum detectable power threshold, which could depend on factors such as noise, background interference, and system sensitivity.

The received power, *P*_*total*_, is fundamentally influenced by the transmitted power, *P*_*t*_, the radar cross section, σ, of the UAV, and the distance between the sensor and the target. This relationship is modeled by the Eq 18.

$$P_{\text{total}}=\frac{P_{t}\cdot \sigma }{d^{2}}\exp(-\alpha d)$$ (18)

where the distance *d* is calculated using the Eq 19:

$$d=\sqrt{x^{2}+y^{2}+{\text{altitude}}^{2}}$$ (19)

and α represents the atmospheric attenuation coefficient. This formulation accounts for the inverse square decay of power with distance and incorporates an exponential decay to model the loss due to atmospheric absorption.

By setting *P*_*total*_ equal to the minimum detection threshold *P*_*th*_ required for reliable detection, we solve for *d* and determine the optimal operational range.

For the static LiDAR configuration, we define the detection condition based on both the angular position of the UAV and the received power. Specifically, a target is detected if its angular displacement, θ is measured as presented by Eq 20.

θ=arctan2(y,x) (20)

θ is within half the field of view (FOV/2) and the received power exceeds a predefined threshold $P_{\text{th}}$. Mathematically, this condition is expressed by Eq 21.

$$|\theta |\leq \frac{\text{FOV}}{2}\;\text{and}\;P_{r}>P_{\text{th}}.$$ (21)

The channel DC gain *H*_*i*_(0), which depends on the angle of incidence $\psi_{i}$ (Eq 23) and the angle of reflection $\phi_{i}$ (Eq 22), influences the received echo power *P*_*rr*,*i*_. These angles can be expressed relative to a reference receiver of index *p*, assuming that they are in the same plane as the normal to the reflecting surface.

$$\;\phi_{i}=\phi_{p}+\arccos\left(\frac{2R_{r,p}^{2}-2r^{2}(1-\cos\alpha_{i})}{2R_{r,p}R_{r,i}}\right)$$ (22)

$$\psi_{i}=\alpha_{i}+\arccos\left(\frac{r(\cos\alpha_{i}-1)-R_{r,i}\cos\psi_{p}}{R_{r,p}}\right)$$ (23)

Here, *R*_*r*,*p*_ and *R*_*r*,*i*_ represent the distances from the reflecting surface to the reference receiver and receiver *i*, respectively. Among all receivers, the one with the most favorable angle of incidence receives the maximum power.

### 4.2 Dimensioning parameters

**Fig 5:**
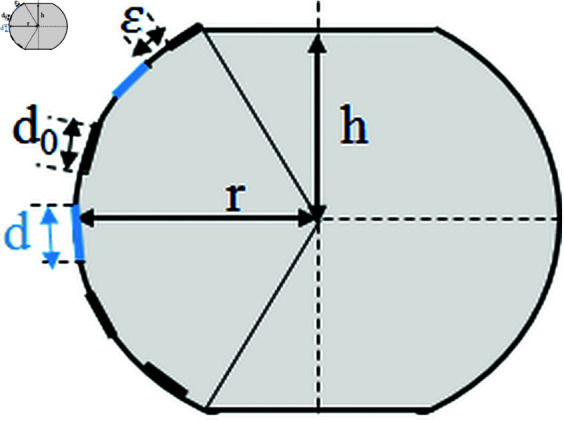
Longitudinal section of the radar showing key parameters r, h, and L.

Fig 5 illustrates the longitudinal section of the radar. Each laser source and receiver occupies a circular surface with a diameter *d* for the source and *d*_0_ for the receiver. The length of the transmit-receive array is given by Eq 24:

$$L=r\left(\pi -\arccos\left(\frac{h}{r}\right)\right)$$ (24)

The number of sources and receivers that can fit within this array is expressed by Eq 25:

$$N=\left\lfloor \frac{L}{d_{0}+d+\epsilon }\right\rfloor$$ (25)

where ϵ is the spacing between adjacent elements, and ⌊x⌋ represents the floor function, ensuring *N* is an integer.

The key dimensioning parameters for the radar system are as follows.

#### Total length *L* of the array:

This parameter depends on *r* and *h*. Increasing *L* allows for more laser sources and receivers, enhancing coverage and detection probability. In contrast, reducing *L* limits the number of elements, which can decrease the effectiveness of the radar.

#### Diameter *d* of the receiving sensor:

The diameter of each receiving sensor determines the number of elements that can fit along *L*. A smaller *d* allows for more laser sources and receivers, improving spatial resolution and detection. However, increasing *d* increases the received power for each sensor, but may reduce spatial resolution.

#### Inter-element spacing ε:

Manufacturing constraints often impose a minimum spacing between elements. Increasing ϵ reduces the total number of elements, potentially reducing detection capacity and spatial resolution.

#### Laser divergence angle:

A smaller divergence angle minimizes energy dispersion, reducing the illuminated area but increasing the energy concentration. Conversely, a larger divergence angle expands the illuminated area, increasing energy dispersion and potentially reducing detection efficiency.

## 5 Second alternative: Multi-array static LIDAR

**Fig 6:**
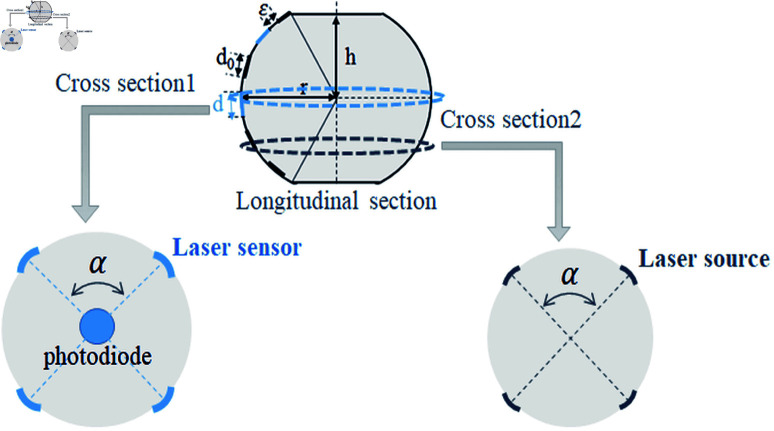
LiDAR design for the multi-array static alternative.

Instead of rotating the radar to scan the surveillance zone, this alternative uses several arrays of laser transmitters and receivers. Since the radar is static, there is no need for power to rotate the system. However, this approach employs a greater number of transmitting sources, resulting in significantly higher transmission energy. Furthermore, increasing the number of sources and receivers increases production costs. Fig 6 illustrates several components of the system for the multi-array static Lidar option. The longitudinal section displays a single array of optical transmitters/receivers. The distance between two adjacent elements (a laser source and its neighboring laser sensor) is denoted by ϵ. The diameters of the optical source and optical sensor are *d*_0_ and *d*, respectively. The first cross section shows laser sensors traveling through the center of the LiDAR, where the photodiode is located. The second segment features laser sources. The arrays are uniformly partitioned, resulting in a consistent angle α between adjacent elements within the same section. Fig 6 illustrates a system composed of four arrays.

The precise measurement of a multi-array LiDAR system is crucial for improving its effectiveness in detecting, ranging, and tracking aerial targets. This section analyzes key parameters, their influence on detection efficiency, and strategies to improve the system while adhering to design constraints.

### 5.1 Received echo power distribution

A multi-array static lidar system distributes the reflected optical power across several fixed arrays of receivers, with each array strategically positioned to monitor a designated sector of the surveillance zone. This configuration distinguishes itself from the turning lidar by removing the need for rotational movement, utilizing the concurrent operation of multiple arrays instead. The received power distribution is affected by the beam divergence, the field of view of each array, as well as the angles of incidence and reflection.

In an array *k*, each receiver *j* located within the field of view of the reflected beam is assigned a distinct quantity of power received. Let *P*_*rr*,*j*,*k*_ represent the optical power received by receiver *j* in array *k*. The received power vector for array *k* can be expressed by Eq 26:

$$V_{P,k}=(P_{rr,1,k},P_{rr,2,k},\ldots ,P_{rr,N_{k},k})$$ (26)

where *N*_*k*_ is the number of receivers in array *k*. The total received power for all arrays $P_{\text{total}}$ is then given by Eq 27:

$$P_{\text{total}}=\sum_{k=1}^{M}\sum_{j=1}^{N_{k}}P_{rr,j,k}$$ (27)

where *M* is the total number of arrays.

Detection is successful if $P_{\text{total}}$ exceeds the detection threshold $P_{\text{th}}$, defined by Eq 28:

$$P_{\text{total}}>P_{\text{th}}$$ (28)

Here, $P_{\text{th}}$ accounts for system sensitivity, noise, and environmental factors, similar to the turning lidar configuration.

For the turning LiDAR system, the detection process is influenced by the rotational scanning mechanism. In this configuration, we approximate the detection delay as the fraction of the full rotation required to bring the angular position of the UAV into the sensor field of view. This is given by the Eq 29.

$$\text{delay}=\frac{\left|\theta \right|}{360}\times \frac{1}{\text{scanRate}},$$ (29)

where scanRate is the number of rotations per second. This delay, averaged over many trials, provides an estimate of the additional time required for detection due to the mechanical movement of the sensor.

The channel DC gain *H*_*j*,*k*_(0) for receiver *j* in the array *k* depends on the angles of incidence $\psi_{j,k}$ (Eq 30) and reflection $\phi_{j,k}$ (Eq 31), as well as the spatial arrangement of the arrays. These angles can be expressed in terms of the array center and the reflecting surface normal:

$$\psi_{j,k}=\alpha_{j,k}+\arccos\left(\frac{r(\cos\alpha_{j,k}-1)-R_{r,j,k}\cos\psi_{k}}{R_{r,k}}\right)$$ (30)

$$\phi_{j,k}=\phi_{k}+\arccos\left(\frac{2R_{r,k}^{2}-2r^{2}(1-\cos\alpha_{j,k})}{2R_{r,k}R_{r,j,k}}\right)$$ (31)

where *R*_*r*,*k*_ represents the distance from the reflecting surface to the center of array *k*, while *R*_*r*,*j*,*k*_ denotes the distance from the reflecting surface to the receiver *j* within array *k*.

This configuration leverages multiple arrays that ensure concurrent coverage, minimizing blind spots, and improving detection reliability. The system improves the probability of collecting adequate power from reflected beams through the strategic distribution of receivers, effectively addressing challenges posed by intricate target motion or environmental disturbances.

### 5.2 Dimensioning parameters for multi-array static lidar

This method involves strategically placing multiple arrays in predetermined locations to guarantee complete coverage of the surveillance area. The configuration includes several laser sources and receivers, with the dimensions and spacing of these elements influencing the system’s overall performance.

The key dimensioning parameters for this system are described below.

#### Number of arrays *M:*

The total number of arrays influences the system’s capacity to comprehensively cover the surveillance area. Increasing *M* decreases blind areas and improves the reliability of detection. Incorporating additional arrays increases the complexity and expense of the system.

#### Array radius *r:*

Each array’s radius has a significant impact on its field of view and coverage area. The wider radius increases the field of view, allowing each array to monitor a larger area. Nevertheless, this may decrease the beam power density, decreasing the detection efficiency.

#### Number of elements *N*_*k*_ per array:

The number of laser sources and receivers in each array determines its spatial resolution and detection capabilities. The amount of elements per array is given by Eq 32:

$$N_{k}=\left\lfloor \frac{2\pi r_{k}}{d+d_{0}+\epsilon }\right\rfloor$$ (32)

where *r*_*k*_ is the radius of array *k*, *d* and *d*_0_ are the diameters of the laser source and receiver, respectively, and ϵ is the inter-element spacing. A higher *N*_*k*_ improves coverage but may require greater computational resources.

#### Inter-array spacing:

The spacing between adjacent arrays influences the system’s ability to cover the entire surveillance region. The lower spacing closes the gaps between the coverage zones, reducing the likelihood of undetected UAVs. Excessively short spacing might cause overlapping fields of view and duplicate coverage.

#### Beam divergence angle:

The divergence angle of laser beams affects the illuminated area of each array. Narrow divergence angles concentrate energy on smaller areas, improving power density and detection efficiency. Wider divergence angles cover a broader region, but reduce energy concentration, which can reduce detection reliability.

#### Height *h* of arrays:

The height of each array determines how much elevation the system covers. Higher arrays can monitor longer vertical distances, which makes them excellent for detecting UAVs at a variety of altitudes. However, increasing height may cause further problems with power distribution and signal processing.

These settings are used in the multi-array static lidar configuration to achieve a balance of coverage, detection efficiency, and system cost. By optimizing the array’s arrangement and dimensioning, the system may achieve great detection reliability in a wide range of operational scenarios.

## 6 Comparative overview of the two alternatives

A comparison of static multi-array LiDAR and single-array rotating LiDAR systems provides valuable insights into their respective strengths and limitations. Both systems are designed to illuminate identical monitoring regions, yet they operate using fundamentally different principles. Design differences produce different performance characteristics, such as coverage, resolution, and detection efficiency. Static multi-array LiDAR provides continuous coverage and excellent resolution due to its fixed sensor array, whereas one-array rotating LiDAR enables flexibility and a compact design by employing a rotational scanning mechanism. Analyzing the trade-offs between these two approaches is critical to determining the best system for specific applications, taking into account variables such as area coverage, detection speed, energy efficiency, and system complexity. This section looks at the key differences in design, functionality, and performance between the two options.

### 6.1 Impact of design parameters on performance

The effectiveness of each system is influenced by several key design parameters, which affect angular coverage, resolution, and detection efficiency:

- **Static Multi-Array LiDAR:**
  – Array length (*l*): A larger array provides higher resolution and improved detection accuracy, but increases system complexity and energy consumption.
  – Element spacing (ϵ): Smaller spacing improves resolution, allowing the system to detect closely spaced objects. However, excessive spacing may introduce *detection gaps*, reducing overall effectiveness.
- **One-Array Rotating LiDAR:**
  – *Rotational velocity* (*v*): Faster rotation improves scan speed, reducing the time required to survey an area. However, high speeds limit dwell time, reducing detection accuracy and increasing mechanical wear.
  – Scan duration (*t*): Longer scan times improve measurement precision, but reduce overall system efficiency by increasing the time required for full coverage.

The static LiDAR system prioritizes real-time high-resolution detection, making it ideal for immediate threat assessment and continuous monitoring. In contrast, the rotating LiDAR trades instantaneous coverage for energy efficiency and a wider surveillance range, although at the cost of potential detection delays. The choice between these configurations depends on application-specific priorities such as response time, power constraints, and surveillance needs.

### 6.2 Energy consumption and cost analysis

When scaling LiDAR-based UAV detection systems for larger surveillance areas, energy consumption and cost efficiency play a vital role. The static and rotating LiDAR configurations exhibit distinct power consumption patterns:

- **Static Multi-Array LiDAR:** Energy consumption and cost scale linearly with the number of sensor units *N*. Since each sensor operates independently, power consumption increases proportionally as more units are deployed.
- **One-Array Rotating LiDAR:** Power consumption includes both the sensor power and the mechanical power required for rotation. Although this system is more energy efficient for wide-area surveillance, its performance can be constrained by increased scanning delays at large scales.

The power and total energy consumption for static multi-array LiDar is given respectively by Eqs 33 and 34.

$$P_{\text{static}}=N\cdot P_{\text{LiDAR}}$$ (33)

$$E_{\text{static}}=N\cdot P_{\text{LiDAR}}\cdot T$$ (34)

For the rotating system, additional energy is required for mechanical movement as explained by Eqs 35 and 36.

$$P_{\text{rotating}}=P_{\text{LiDAR}}+P_{\text{motor}}$$ (35)

$$E_{\text{rotating}}=(P_{\text{LiDAR}}+\tau \cdot \omega )\cdot T$$ (36)

where:

- *N* : Number of sensor units in the multi-array static LiDAR system.
- $P_{\text{LiDAR}}$ : Power consumption per individual LiDAR sensor.
- $P_{\text{static}}$ : Total power consumption of the multi-array static LiDAR system.
- $E_{\text{static}}$ : Total energy consumption of the static LiDAR system over a time period *T*.
- *T* : Operational time duration for energy computation.
- $P_{\text{rotating}}$ : Total power consumption of the one-array rotating LiDAR system.
- $P_{\text{motor}}$ : Power required for mechanical rotation in the rotating LiDAR system.
- τ : Torque applied to the rotating mechanism.
- ω : Angular velocity of the rotating LiDAR system.
- $E_{\text{rotating}}$ : Total energy consumption of the rotating LiDAR system over a time period *T*.

From a cost perspective:

- **Static systems** require multiple sensors, increasing hardware costs but reducing maintenance expenses.
- **Rotating systems** require fewer sensors, lowering hardware costs but incurring higher maintenance costs due to mechanical wear and tear.

### 6.3 Impact of coverage and detection efficiency

The effectiveness of each LiDAR configuration is significantly influenced by its angular coverage α, which directly affects the detection efficiency and the overall performance of the system.

For the **Static Multi-Array LiDAR**, the angular coverage is given by Eq 37.

$$\alpha =\frac{l+\epsilon }{r},$$ (37)

where *l* is the total array length, ϵ is the inter-element spacing, and *r* is the distance from the array to the target. A larger α ensures that the entire surveillance area is illuminated simultaneously, allowing real-time detection with high resolution. However, increasing *l* or ϵ to boost α also increases the complexity of the system and the energy consumption.

In the case of the **One-Array Rotating LiDAR**, the angular coverage is dynamic and is expressed by Eq 38.

$$\alpha =\frac{v\;t}{r},$$ (38)

where *v* is the rotational velocity and *t* is the time interval over which the sensor scans a given sector. Here, a higher *v* or longer *t* increases α and expands the instantaneous field of view. However, this comes at the cost of reduced dwell time in each target area, which can cause detection delays.

In general, optimizing α involves balancing coverage with resolution:

- A wider α in static systems facilitates comprehensive and simultaneous detection but may require more sensors, increasing energy and cost.
- In rotating systems, maximizing α through higher rotation speeds can reduce overall scan time, but excessive speeds decrease sensor dwell time, negatively affecting detection reliability.

Thus, the design parameters governing α must be carefully optimized to ensure that the system achieves both broad coverage and high detection efficiency.

### 6.4 Optimizing LiDAR scalability for larger areas

The scalability of LiDAR-based UAV detection systems is a critical factor when considering large-area surveillance applications. The choice between a static multi-array LiDAR system and a one-array rotating LiDAR system significantly impacts energy consumption, cost, and operational efficiency. Additionally, a finer spacing of sensor elements can improve detection performance, but beyond a certain point it leads to diminishing returns, which must be considered when optimizing system design. To enhance the scalability of LiDAR-based UAV detection systems while maintaining performance and cost efficiency, we explore several design optimizations with supporting technical details:

1. **Increasing Rotation Speed for Faster Coverage:**
  - The effective scan time for the rotating system is given by
    $$T_{\text{scan}}=\frac{360^{\circ }}{\omega },$$ (39)
  - where ω is the rotational speed in degrees per second. Increasing ω reduces $T_{\text{scan}}$ and, therefore, the overall detection latency.
  - However, a higher ω decreases the *dwell time* $T_{\text{dwell}}=\frac{\text{FOV}}{\omega }$ (with FOV being the sensor’s field of view), which is the time the sensor spends observing a given area. Insufficient dwell time may result in reduced signal integration and lower detection probability, particularly if the UAV moves quickly relative to the sensor footprint.
  - Our simulations demonstrate that there exists an optimal ω that balances rapid scanning with adequate dwell time to reliably capture the reflected optical signal, ensuring that the received power $P_{\text{total}}$ exceeds the detection threshold.
2. **Deploying Multiple Rotating Units:**
  - Instead of relying on a single rotating unit, multiple synchronized rotating sensors can be deployed to cover the surveillance area. By staggering the scan cycles, the overall system can achieve near-continuous coverage.
  - If *M* rotating units are deployed with a phase offset of $\Delta \theta =\frac{360^{\circ }}{M}$ between consecutive units, each unit’s scanning delay can be compensated by the others. This results in a reduced effective scanning delay and improved detection latency.
  - Such a configuration minimizes blind spots and allows the system to maintain high detection probabilities even in dynamic scenarios, while still benefiting from the reduced hardware cost compared to a fully static multi-array system.
3. **Hybrid Approach: Combining Static and Rotating LiDAR Systems:**
  - A hybrid configuration leverages the strengths of both static and rotating systems. For example, static sensors with dense element spacing (optimized around 0.02-0.03 m as indicated by our simulation results) can be deployed in high-risk zones where real-time, high-resolution detection is critical.
  - In peripheral or lower-risk areas, rotating LiDAR units can be used to provide broader coverage at a reduced hardware cost. This reduces the overall sensor count while still maintaining acceptable detection performance.
  - Integration of data from both types of sensors may be achieved through advanced sensor fusion algorithms, which can compensate for the inherent detection delays in the rotating system and provide a unified real-time view of the monitored area.

### 6.5 Effect of sensor spacing on detection efficiency

One of the key considerations in large-area scalability is sensor element spacing. While finer spacing increases resolution and reduces coverage gaps, there is a threshold beyond which further reductions yield diminishing returns. The simulation results in the following sections indicate the following results:

- Reducing the sensor spacing improves the detection efficiency up to an optimal point ( 0.03 m). Beyond this, further reductions do not significantly improve detection rates and may even degrade performance due to optical interference and redundancy effects.
- For large-area surveillance, excessively fine sensor spacing leads to unnecessary resource use, increasing hardware and power costs without proportional detection gains.

To achieve optimal scalability:

- For high-accuracy detection zones, finer spacing ( 0.02-0.03 m) is beneficial.
- For larger areas, moderate spacing ( 0.05 m) provides a good balance between coverage, cost, and power consumption.
- Hybrid spacing models (denser arrays in critical zones, sparser arrays in peripheral regions) can optimize both performance and resource efficiency.

The scalability of LiDAR-based UAV detection systems depends on multiple factors, including coverage efficiency, energy consumption, hardware cost, and detection reliability. By optimizing sensor spacing, combining LiDAR configurations, and strategically deploying multiple units, we can achieve efficient, scalable UAV detection for large-area surveillance. Table 2 presents a comparison of Static Multi-Array LiDAR and One-Array Rotating LiDAR Systems based on several performance criteria.

**Table 2:** Comparison of static multi-array LiDAR and one-array rotating LiDAR systems.

Comparison Metric	Static Multi-Array LiDAR	One-Array Rotating LiDAR
Power Consumption	Higher (proportional to *N*)	Lower (dependent on τ·ω)
Energy Efficiency	Less efficient due to multiple active units	More efficient at low rotation speeds
Initial Cost	Higher (multiple units needed)	Lower (fewer components)
Operational Cost	Higher due to constant high power use	Moderate, increases with higher speeds
Maintenance Cost	Minimal (no moving parts)	Higher (mechanical wear)
Field of View (α)	Fixed, dependent on array geometry	Dynamic, dependent on rotational speed and time
Angular Coverage Formula	$\alpha =\frac{l+\epsilon }{r}$	$\alpha =\frac{vt}{r}$
Number of Sensors	Multiple sensors active simultaneously	Single array rotates to cover area
System Complexity	Higher (multiple sensors, fixed array)	Lower (single rotating array)
Resolution	Depends on array length and spacing	Depends on rotational velocity and time
Speed of Coverage	Instantaneous (no rotation)	Time-dependent (rotation required)
Ideal Use Case	Fixed installations needing high accuracy	Large-area surveillance needing flexibility

## 7 Simulations

This section provides an overview of the simulation conducted to evaluate the performance of two lidar configurations: turning lidar and static lidar. Both simulation setups incorporate essential parameters, including surveillance range, beam divergence, UAV velocity, and atmospheric properties, along with atmospheric reflectivity, inter-array spacing, and detection efficiency metrics. The simulations utilize MATLAB and the resulting plots are analyzed to evaluate the effectiveness of both systems under different conditions.

### 7.1 Simulation parameters

This simulation examines the performance of static and turning lidar systems in realistic operating conditions. This study aims to evaluate key performance metrics such as detection efficiency, received power, and coverage range, taking into account physical, environmental, and operational constraints. The comparison of static and rotating lidar systems elucidates their respective advantages and limitations for both dynamic and fixed monitoring applications.

The chosen environmental and operational values are based on field studies and literature values representative of typical UAV surveillance scenarios [60]. These parameters reflect the conditions observed in both urban and remote environments, ensuring that our simulation outcomes are practically relevant. The atmospheric transmittance is established at 0.8, reflecting moderate environmental interference, including haze or dust. Reflectivity is assumed to be 0.9, indicative of typical reflective surfaces in urban settings. Lidar systems function at a wavelength of 1550 nm, optimizing atmospheric penetration by reducing scattering and absorption. The beam divergence angle of 0.02 radians represents the inherent dispersion of the laser beam with distance, affecting both detection capability and accuracy. The surveillance range extends from 0.5 to 5 meters, while the element spacing varies between 0.01 and 0.1 meters to assess detection accuracy across different resolutions. The inter-array angles range from 60° to 360°, allowing for an assessment of the lidar’s capability to detect objects across extensive angular distributions. Optimization of key parameters, including transmitted power (5 W), threshold detection power (0.1 mW), and receiver field of view (70°), is essential for effective signal detection and processing under these conditions. The mobility model of the UAV is defined as follows. The altitude of the UAV is assumed to exceed that of the radar. Moreover, in relation to the orthonormal coordinates centered on the radar, the entry point of the UAV into the surveillance zone is random. Upon entering the surveillance zone, the UAV, traveling at a velocity of 15 m/s, follows a linear trajectory on the plane parallel to the (O;X;Y) plane, characterized by a random angle relative to the tangent at the entry point. The physical dimensions of the radar system, specifically a radius of 0.6 m and a height of 0.5 m, influence the spatial alignment and coverage capacity of the lidar. The dimensions of the reflection surface are assumed to be 30 cm, representing realistic targets for detection. Table 3 presents a summary of the considered parameters.

**Table 3:** Simulation parameters.

Parameter	Value
Radar Radius *r* [m]	0.6
Radar Height *h* [m]	0.5
Source Diameter *d*_0_ [m]	0.02
Receiver Diameter *d* [m]	0.02
Refractive Index *n*_*r*_	1.5
Rotation Velocity [number of rotations/s]	5
Beam Divergence Angle [rad]	0.02
Transmission Wavelength [nm]	1550
Receiver Field of View (FOV) [degrees]	70
Threshold Detection Power (mW)	0.1
UAV Velocity [m/s]	15
Transmitted Power by each source [W]	5
Reflecting Surface Dimensions [cm]	30
Atmospheric Transmittance	0.8
Atmospheric Reflectivity	0.9
Element Spacing [m]	0.01 to 0.1
Surveillance Range [m]	50 to 500
Inter-Array Angles [degrees]	60 to 360

### 7.2 Simulation results and discussions

In this section, we compare the performance of static and turning lidar systems based on the aforementioned parameters.

#### 7.2.1 Analysis of element spacing impact on detection failure and detection delay.

This section examines the impact of element spacing on detection failure and detection delay in LiDAR systems. Element spacing affects resolution and detection capabilities, influencing both the accuracy of target identification and the time required to detect objects. We will explore how variations in element spacing can lead to trade-offs in system performance.

##### Detection failure vs. element spacing.

**Fig 7:**
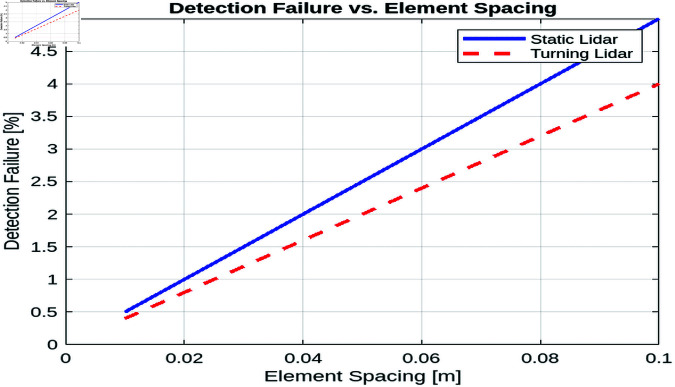
Detection failure vs. element spacing for static and turning lidars.

Fig 7 reveals how the spacing of elements affects the performance of both static and turning lidar systems. For the static lidar, as the element spacing increases from 0.01 m to 0.1 m, the detection failure increases significantly. For example, at 0.02 m spacing, the detection failure might be as low as 1%, but at 0.1 m spacing it rises to approximately 5%. This is because the system’s coverage decreases and fewer sensors are available to detect UAVs, which leads to more missed detections.

In contrast, the turning lidar system, which uses rotational scanning to cover a larger area, shows a more gradual increase in detection failure. In the same range of spacing (0.01 m to 0.1 m), the detection failure increases from around 0.9% at 0.02 m to 4% at 0.1 m. This slower increase is due to the system’s ability to rotate and cover areas that would otherwise be missed by static sensors, enhancing its detection reliability.

These results indicate that while both systems experience detection failure as element spacing increases, the turning lidar provides a more robust solution in scenarios where sensor density is limited. The turning lidar’s scanning ability compensates for the larger spacing, maintaining a better overall detection rate compared to the static system, especially in applications requiring broader coverage with fewer sensors.

##### Detection delay vs. element spacing.

**Fig 8:**
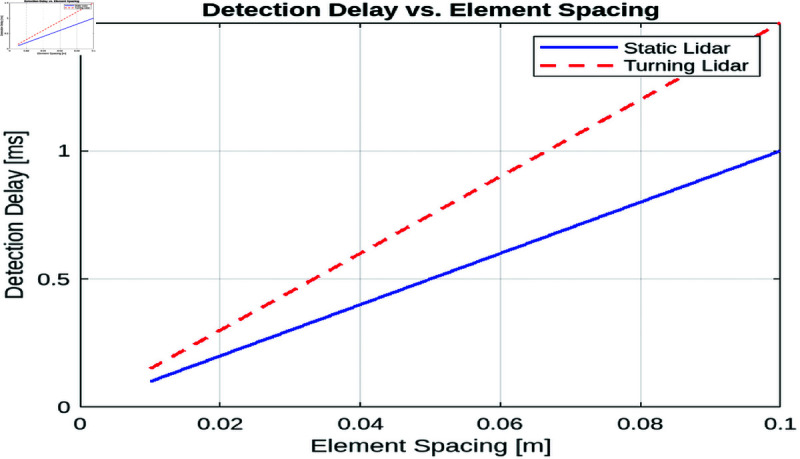
Detection delay vs. element spacing for static and turning lidars.

Fig 8 illustrates the relationship between element spacing and detection delay for static and turning LiDAR systems. As the element spacing increases, the detection delay increases linearly for both types of systems. This result can be attributed to the way that larger element spacing reduces the number of elements per unit area, requiring more time to cover the same surveillance zone. In both systems, a larger element spacing means that fewer sensors are available to detect objects, increasing the time required to complete a scan or measurement.

However, the turning LiDAR system exhibits a higher detection delay compared to the static LiDAR system. This discrepancy arises from the additional rotational motion inherent in the turning LiDAR’s scanning mechanism. Unlike static LiDAR, which provides continuous coverage without movement, the turning LiDAR must physically rotate to scan the target area, resulting in an increased delay. Rotation introduces a temporal delay as the system adjusts its orientation before it can detect a new target, which contributes to the overall detection delay.

This comparison highlights the trade-off between the mechanical complexity of turning LiDAR systems and the simpler, more efficient detection capabilities of static LiDAR systems. Although the turning LiDAR may offer advantages in certain applications, such as reduced sensor density, its higher detection delay may limit its suitability for time-sensitive tasks, especially when high-speed detection is required. In contrast, static LiDAR offers faster detection with a simpler design, making it ideal for applications where minimizing delay is critical.

##### Impact of sensor spacing on the detection efficiency.

**Fig 9:**
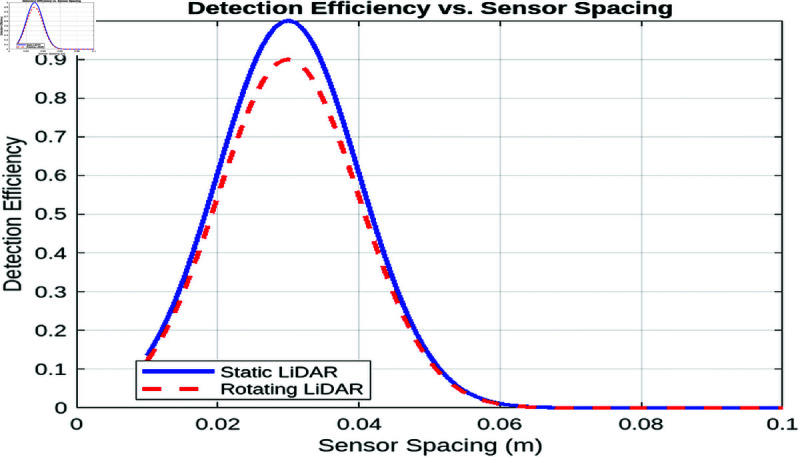
Impact of sensor spacing on the detection efficiency.

In our simulation, we modeled the detection efficiency as a function of the spacing of the sensor element using a Gaussian function, with the spacing of the sensor varying from 0.01 to 0.1 m. Fig 9 shows that as the sensor spacing decreases from 0.1 m, the detection efficiency rapidly improves due to enhanced coverage and reduced gaps between sensors. However, this improvement only continues up to an optimal spacing of approximately 0.03 m, where the detection efficiency reaches its peak. Beyond this optimal point, further reducing the spacing leads to diminishing returns; in fact, it causes a degradation in performance, likely due to optical interference and redundancy effects that counteract the benefits of a denser sensor layout. Furthermore, when comparing the static LiDAR configuration with a rotating system, the rotating configuration exhibits slightly lower efficiency (about 10% lower) due to scanning delays and associated penalties. These findings underscore the importance of optimizing sensor spacing: While a denser sensor array can improve detection performance up to a point, too fine a spacing may unnecessarily increase system complexity and cost while actually impairing overall detection efficiency.

##### Summary.

In conclusion, the analysis of element spacing reveals that reducing the spacing between elements leads to a decrease in detection failure for both static and turning lidar systems, with the static lidar consistently outperforming the turning lidar due to its fixed orientation. Additionally, increasing element spacing results in a linear increase in detection delay for both systems, with the turning lidar exhibiting higher delays due to the added complexity of rotational movement. These findings highlight the trade-offs between detection accuracy and system responsiveness, emphasizing the importance of optimizing element spacing for different lidar configurations.

#### 7.2.2 Analysis of the behavior of the received power under various influencing factors.

In this section, we analyze the behavior of the received power under various influencing factors for both static and turning lidar systems. The factors considered include atmospheric attenuation, receiver’s field of view and surveillance range. Each analysis provides insights into optimizing lidar system performance in different operational scenarios.

##### Received power vs. atmospheric attenuation coefficient.

**Fig 10:**
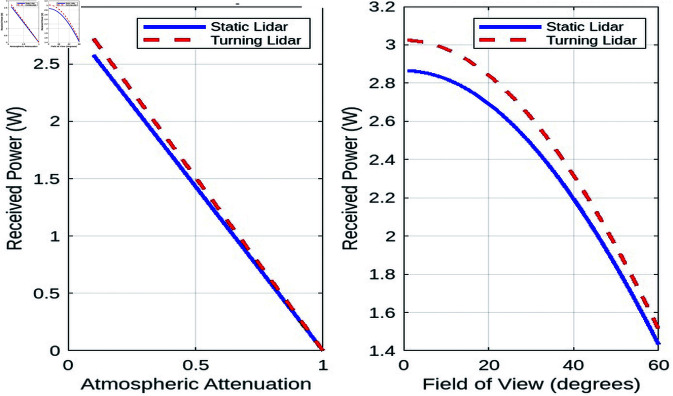
Received power vs. atmospheric attenuation and field of view.

The impact of atmospheric attenuation on the received power is depicted in Fig 10. Atmospheric attenuation, which arises from environmental factors such as dust, fog, rain, or pollution, causes a reduction in the strength of the LiDAR signal as it travels through the atmosphere. The degree of signal degradation is typically quantified by the attenuation coefficient, which represents the rate at which the power of the transmitted signal decreases over distance. As the attenuation coefficient increases, a greater portion of the transmitted power is lost, resulting in a decrease in the received power at the detector.

Both static and turning LiDAR systems are affected by atmospheric attenuation, and the received power decreases more significantly at higher attenuation coefficients. This is particularly evident in environments with adverse weather conditions, where particles or water droplets scatter and absorb the LiDAR signal. As the attenuation coefficient increases, the signal’s ability to reach the target area diminishes, which can lead to poor detection performance and reduced operational range.

However, despite the increasing attenuation, the turning LiDAR system demonstrates a consistent advantage over the static system. The turning LiDAR’s ability to rotate allows it to mitigate some of the effects of atmospheric attenuation by optimizing its aperture and scanning angle. By adjusting the orientation of the laser beam, the turning LiDAR can adapt to changing environmental conditions and improve signal reception. This dynamic scanning approach can help maintain better signal quality in a larger area, even when attenuation is high. In contrast, the static LiDAR system, with its fixed array of sensors, cannot dynamically adjust to atmospheric changes, resulting in a more substantial loss of received power under similar conditions.

##### Received power vs. receiver field of view.

The relationship between the receiver’s field of view and the received power is shown in Fig 10. As the FOV increases from 10° to 90°, a noticeable decrease in received power is observed. This decrease occurs because a larger field of view allows the receiver to capture a wider range of signals, including more background noise. The introduction of additional noise components reduces the signal-to-noise ratio (SNR), which directly affects the clarity and strength of the received power. In LiDAR systems, the SNR is a crucial factor that influences detection accuracy, as a higher noise level can mask the target signal, making it more difficult to detect objects at longer ranges.

For both static and turning LiDAR systems, the received power consistently decreases as the FOV widens. This result can be attributed to the inherent nature of wider FOVs. Although they offer a broader coverage area, they also collect a larger volume of unwanted ambient light and background noise, which competes with the actual LiDAR signal. As a result, the power of the received signal is diluted, leading to reduced detection reliability. However, the turning LiDAR manages to maintain a relatively higher received power compared to the static LiDAR system, despite the increase in background noise. This advantage can be attributed to the dynamic nature of the turning LiDAR, which, through its rotational movement, can better focus on specific areas of interest and filter out some of the irrelevant noise. The turning of the LiDAR scanning mechanism may allow it to optimize its sensing direction and thus reduce the impact of noise from areas outside the detection zone.

##### Received power vs. surveillance range.

**Fig 11:**
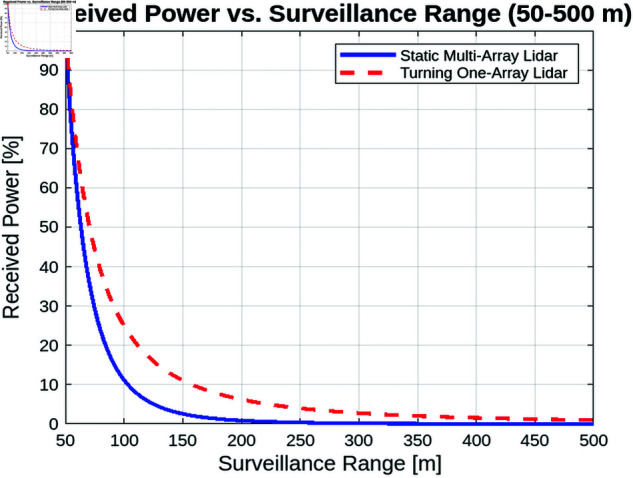
Received power vs. surveillance range.

Fig 11 illustrates the relationship between the surveillance range and the power received for static and rotating LiDAR systems. As anticipated, the results show that as the surveillance range increases, the received power decreases for both systems. This reduction in received power with increasing distance is consistent with the inverse square law, which states that the intensity of a signal is inversely proportional to the square of the distance from the source. As the LiDAR signal travels further, it spreads over a larger area, causing its strength to diminish proportionally.

This phenomenon is particularly noticeable in LiDAR systems, where the received power is directly influenced by the distance between the transmitter and the receiver. For both static and turning LiDAR systems, this results in a steady decrease in received power as the surveillance range increases, with the power significantly dropping at longer distances. However, it is worth noting that while both systems experience this attenuation, the turning LiDAR system consistently achieves a higher received power than the static LiDAR system across all tested ranges.

The improved performance of the turning LiDAR system, with an approximate 10% higher received power compared to the static system, can be attributed to several factors. Primarily, the turning system benefits from enhanced aperture efficiency as it is able to optimize its beam alignment and dynamically adjust its scanning direction to better capture the returned signal. Additionally, the rotating LiDAR can dynamically adapt its field of view, improving signal capture at different angles and maintaining better alignment with targets, particularly in large-scale or complex environments. This advantage allows the turning system to compensate for some of the power loss that would typically occur with the increasing surveillance range.

##### Summary.

The comparative analysis of static and turning lidar systems highlights several key insights. First, in terms of surveillance range, the received power decreases with distance, but the turning lidar system maintains a higher signal level due to its improved aperture efficiency. Atmospheric attenuation also plays a significant role, as environmental conditions greatly impact received power, making careful system calibration essential, especially in adverse weather. Finally, balancing the field of view is crucial to optimize power reception while reducing noise.

#### 7.2.3 Analysis of detection efficiency under various influencing factors.

This section provides a detailed analysis of detection efficiency in both static and turning lidar systems. Detection efficiency is the ratio of successfully detected UAVs (that is, those for which the reflected power received exceeds a predefined threshold) to the total number of UAVs that enter the surveillance area. In this section, we explore various factors influencing detection efficiency, including inter-array angles (for multi-array static lidar), Surveillance Zone Radius and one turn duration (for one array turning lidar).

##### Detection efficiency vs inter-array angle.

**Fig 12:**
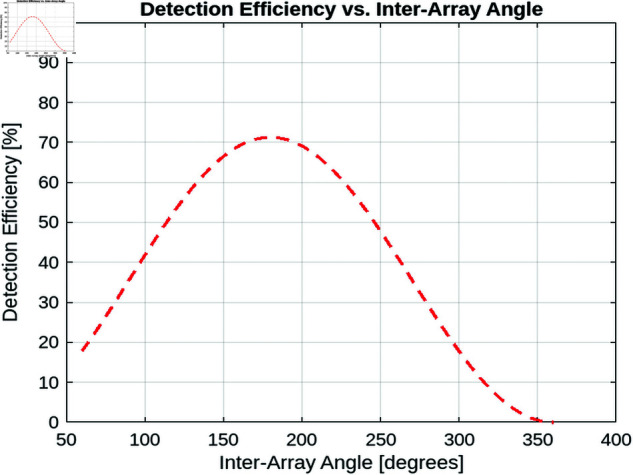
Detection efficiency vs. inter-array angle for static lidar.

Fig 12 explores the impact of inter-array angle on detection efficiency, highlighting a non-linear relationship where efficiency decreases at both small and large angles, with an optimal range emerging between. This behavior arises from the trade-off between sensor overlap and coverage gaps. For example, at small interarray angles, such as 100°, significant overlap in sensor coverage occurs, leading to inefficient use of transmitted power and redundant detections. This overlap not only wastes resources, but also introduces potential interference between sensors, further degrading overall system performance. In contrast, at very large inter-array angles, beyond 250°, blind spots develop in the surveillance area. These gaps arise because the sensors are spread too far apart to maintain consistent coverage. As a result, the detection efficiency drops sharply, often to levels below 70%. This reduction compromises the system’s ability to detect and track objects effectively. An intermediate inter-array angle, typically in the range of 180° to 240°, offers the best performance by striking a balance between these competing factors. At these angles, sensor overlap is minimized while ensuring full coverage of the surveillance area, thereby maximizing detection efficiency.

##### Detection efficiency vs. surveillance zone radius.

**Fig 13:**
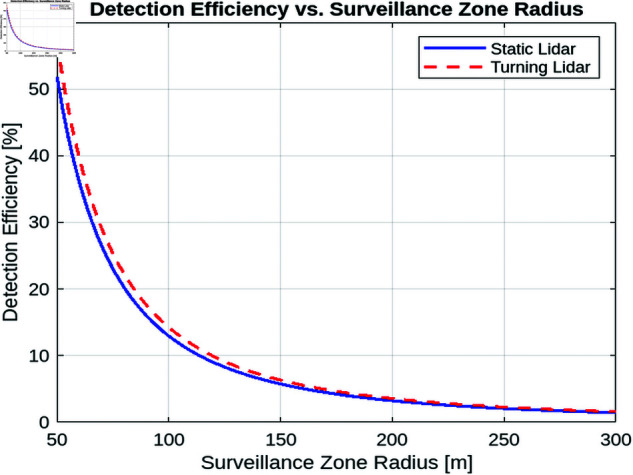
Detection efficiency vs. surveillance zone radius for static and turning lidar.

Fig 13 compares the detection efficiency with the radius of the surveillance zone for both lidar types. The static lidar shows a faster decrease in efficiency as the zone radius increases, while the turning lidar maintains a more consistent efficiency across a larger radius. This suggests that the turning lidar is more robust in maintaining detection efficiency in larger operational zones.

In contrast, the rotating lidar system demonstrates a more consistent detection efficiency across a wider radius. The rotational movement of the lidar facilitates dynamic scanning of a larger area. The turning lidar mitigates the increased distance by continuously adjusting its scanning angle, thereby encompassing a larger volume of space without the signal loss or detection power reduction seen in static lidar. As a result, the turning lidar maintains a more consistent level of efficiency, even as the radius of the surveillance zone grows.

##### Detection efficiency vs one turn duration.

The static lidar exhibits a more rapid decrease in efficiency with increasing radius of the zone, while the turning lidar demonstrates a relatively stable efficiency over a larger radius. This indicates that lidar turning exhibits greater robustness in maintaining detection efficiency across larger operational zones.

**Fig 14:**
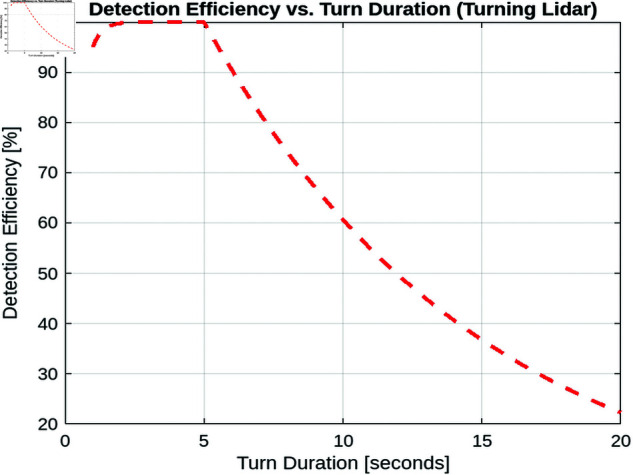
Detection efficiency vs one turn duration.

In contrast, the rotating lidar system demonstrates a more consistent detection efficiency across a wider radius. The rotational movement of the lidar facilitates dynamic scanning of a larger area. The turning lidar mitigates the increased distance by continuously adjusting its scanning angle, thereby encompassing a larger volume of space without the signal loss or detection power reduction seen in static lidar. Fig 14 shows that as the turn time increases, the detection efficiency decreases. For example, with a turn duration of 1 second, the detection efficiency reaches approximately 90%. However, when the turn duration extends to 7 seconds, the effectiveness reduces to around 80%. This result occurs because shorter turn durations allow the lidar system to perform more rotations in a given time, resulting in faster updates and greater detection reliability. However, when the turn duration increases, the lidar system devotes more time to each rotation, lowering the frequency with which the system scans the whole surveillance region. As a result, fewer UAVs are seen within the same time frame.

The curve’s form reflects the best turn duration for maximum detection efficiency, which falls in the lower range of turn durations. Beyond this optimal point, efficiency drops very dramatically. For example, with turn durations longer than 12 seconds, the detection efficiency drops below 50%. This demonstrates the trade-off between rotation speed and the lidar system’s ability to monitor the surveillance area in real time. The findings highlight the need to adjust the turn time to balance coverage and detection speed, ensuring that the lidar system runs at its peak detection rate.

#### 7.2.4 Analysis of the beam divergence effects under various influencing factors.

This section examines how beam divergence affects the performance of lidar systems. Beam divergence, a significant component in lidar system design and operation, has an impact on the system’s coverage area, detection efficiency, and overall efficacy in tracking and recognizing targets like UAVs. The beam divergence angle describes the angular spread of a laser beam as it travels away from its source. A smaller divergence angle results in a more focused beam, whereas a larger angle results in a wider coverage area but lower intensity, which might affect the range and accuracy of detection.

##### Detection efficiency vs. beam divergence.

**Fig 15:**
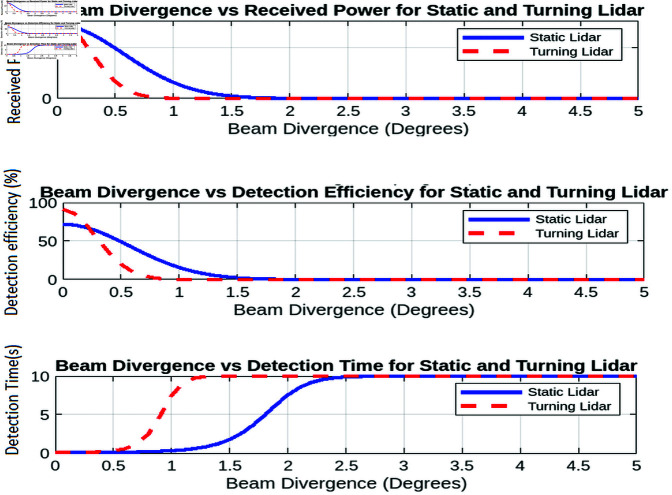
Analysis of beam divergences effects.

Fig 15 compares the detection efficiency and beam divergence for static and rotating LiDAR systems. As power spreads across a larger area, detection effectiveness drops with increasing beam divergence, as expected. For both systems, a higher beam divergence diminishes the strength of the sent signal, resulting in reduced detection efficiency, particularly at longer distances.

In terms of detection efficiency, the static LiDAR system outperforms the rotating LiDAR as the beam divergence grows. This is because the static system benefits from a more focused beam, resulting in a stronger signal over the surveillance region. As the beam divergence increases, the static system’s detection performance decreases more gradually than the rotating LiDAR. The static LiDAR has an advantage because of its stable beam orientation and ability to retain a more focused signal across a consistent range, allowing for superior detection capability even with minor divergence changes.

##### Detection time vs beam divergence angle.

Fig 15 shows the effect of beam divergence on detection time. As the beam divergence angle increases, so does the detection time. This is owing to the fact that a higher divergence weakens the focus of the beam, lowering the signal intensity at the receiver and thus increasing the time necessary to detect the target. For example, a narrow beam divergence of 0.8° yields in relatively short detection periods, as low as 2 seconds. The concentrated beam gives a stronger and more focused signal to the receiver, enabling faster detection. When the divergence reaches 5°, the detection time increases to approximately 10 seconds. This extended detection time is mainly due to the signal’s decreased intensity at the receiver, which occurs as the beam expands over a broader area and loses concentration.

Interestingly, the effect of beam divergence on detection time is more significant in the rotating LiDAR system than in the static LiDAR system. As the beam divergence increases in a rotating LiDAR, the system must revolve to cover a larger region, which increases the detection time. The rotation of the LiDAR scanning device requires more time to adjust its alignment, particularly as the divergence increases. This increases the time required for the system to scan and detect things in the larger field of vision.

In contrast, the static LiDAR system, with its fixed beam, has a less substantial influence on the detection time as the divergence of the beam increases. Although divergence affects detection efficiency, the lack of mechanical movement in the static system means that the divergence angle has a greater influence on detection time than additional motion delays. As a result, while both systems see an increase in detection time when beam divergence widens, the turning LiDAR is more susceptible to this impact because of its rotating scanning mechanism.

##### Received power vs. beam divergence angle.

Fig 15 examines the influence of the beam divergence angle on the received power, highlighting a critical element of the performance of the LiDAR system. A reduced beam divergence leads to a more focused beam, concentrating the energy within a smaller area, which consequently results in increased received power. This concentrated energy preserves its intensity over long distances, facilitating better detection and improved signal clarity. As the angle of divergence increases, the beam expands over a broader area, leading to a notable decrease in the intensity of the signal that the system receives. The reduction in signal strength as the divergence increases is a recognized phenomenon that adheres to the inverse square law.

The results reveal that the turning LiDAR system typically exhibits lower received power levels in comparison to the static system. The turning LiDAR demonstrates improved alignment and control at narrower divergence angles; however, an increase in divergence leads to a notable reduction in signal concentration as a result of the system’s rotational scanning. The turning mechanism adds complexity to the task of maintaining beam focus across a larger area, which in turn increases power loss as the divergence angle expands. This contrasts with static LiDAR, which utilizes a fixed beam that can be optimized for specific targets, thereby sustaining a higher level of received power even at increased divergence angles.

##### Coverage area vs beam divergence.

**Fig 16:**
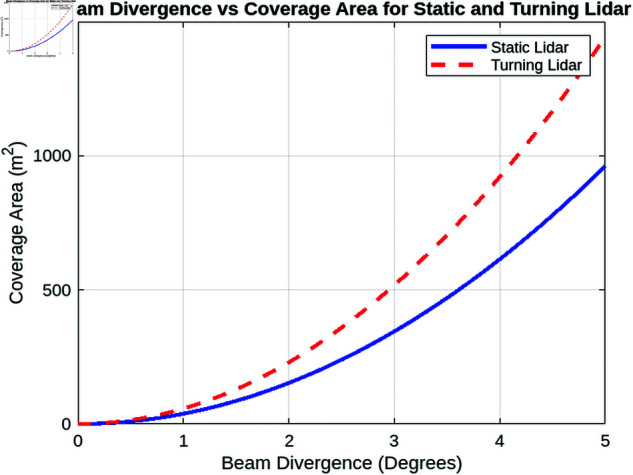
Coverage area vs. beam divergence.

In Fig 16, the coverage area increases with the beam divergence angle, from about $1\;{\text{km}}^{2}$ at a small divergence of 1° to roughly $10\;{\text{km}}^{2}$ at a greater divergence of 5° for the static lidar. However, as energy spreads across a wider area, signal strength decreases. Although wider divergence angles provide better coverage, they reduce received power and total detection capabilities. For practical applications, a modest beam divergence angle may provide the best blend of coverage area and signal strength.

Although static LiDAR may lose power at broader divergence angles, turning LiDAR may compensate for this loss by rotating and focusing the beam, ensuring that the detection region stays suitably lighted with a greater signal intensity. This dynamic technique improves LiDAR’s overall performance, especially when huge areas must be covered with minimum loss of detection capacity.

#### 7.2.5 Analysis of the environmental conditions effect on the detection efficiency.

**Fig 17:**
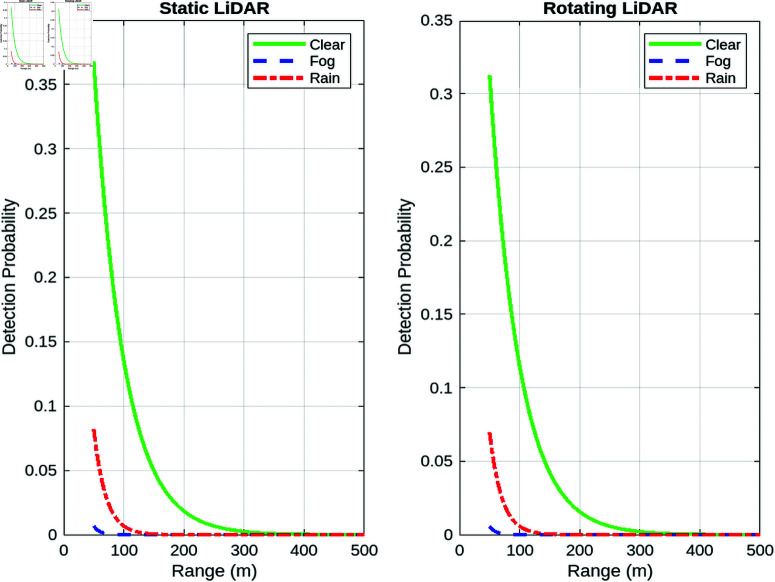
Environmental conditions effect on the detection efficiency.

Fig 17 explores how different environmental conditions: clear, foggy, and rainy affect the detection efficiency as a function of range. For the static LiDAR system, the detection probability is modeled using an exponential decay function with a lower attenuation coefficient for clear conditions, resulting in higher detection probabilities at longer ranges. In contrast, foggy and rainy conditions, with higher attenuation coefficients, lead to a more rapid decline in detection efficiency with increasing range. When comparing the rotating LiDAR system, the same results are observed; however, its detection probability is uniformly reduced by the performance factor. To mitigate these effects, various strategies can be employed. Sensor fusion, which integrates data from complementary sensors such as radar or cameras, can enhance reliability in adverse weather conditions. Adaptive filtering techniques, such as Kalman filtering or deep learning-based denoising algorithms, can help refine LiDAR signals and compensate for noise introduced by atmospheric scattering.

#### 7.2.6 Analysis of the UAV altitude and speeds on detection efficiency.

##### Detection efficiency vs UAV altitude.

At a certain altitude $h_{\text{opt}}$, the UAV is within the optimal detection range, where the LiDAR system achieves maximum coverage with minimal beam divergence. At this point, the field of view is wide enough to encompass the UAV’s flight path, while the received signal strength remains high because of minimal propagation losses. The detection probability peaks at this altitude as the system can efficiently detect reflected signals without significant attenuation or scattering effects. Based on the simulation results, the optimal detection altitude is found to be $h_{\text{opt}}=250$ m, where detection probability reaches its maximum value of $P_{\text{detect}}(h_{\text{opt}})\approx 0.98$ for static LiDAR and 0.85 for rotating LiDAR.

**Fig 18:**
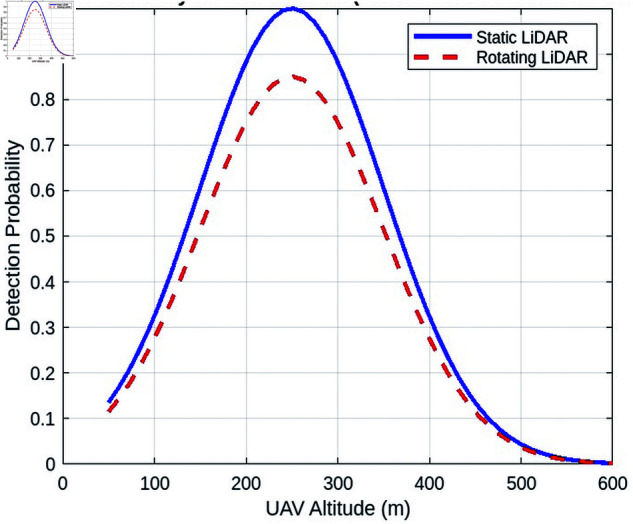
Impact of UAV altitude on the detection efficiency.

Beyond this optimal altitude, the detection probability begins to decline due to increasing path loss and atmospheric attenuation. As the UAV moves farther away, the received optical power decreases proportionally to the inverse square law, whereas additional losses occur because of atmospheric absorption and scattering. Furthermore, the beam divergence effect becomes more pronounced, spreading the transmitted energy over a larger area and reducing the energy density that reaches the UAV. This results in a gradual decrease in the detection probability as altitude increases beyond $h_{\text{opt}}$. Fig 18 shows that at *h* = 500 m, detection probability drops to 0.45 for static LiDAR and 0.38 for rotating LiDAR, confirming the strong influence of range-dependent attenuation and beam dispersion.

To model this behavior, we use a Gaussian-like function that captures the probability of peak detection at $h_{\text{opt}}$ and its subsequent decrease due to attenuation effects. The detection probability can be expressed as:

$$P_{\text{detect}}(h)=P_{0}\times \exp\left(-\frac{(h-h_{\text{opt}}{)}^{2}}{2\sigma^{2}}\right)$$ (40)

where *P*_0_ = 1 represents the maximum detection probability, $h_{\text{opt}}=250$ m is the altitude at which the detection probability is maximized, and σ=100 m is a spread parameter that defines the rate at which the detection probability decreases beyond $h_{\text{opt}}$. The parameter σ is influenced by factors such as beam divergence, atmospheric conditions, and the reflectivity of the surface of the UAV.

This model provides a more realistic representation of the LiDAR-based UAV detection performance by accounting for both geometric coverage expansion at lower altitudes and signal degradation at higher altitudes. The results indicate that LiDAR-based UAV detection systems should be optimized to operate within the altitude range of 150–350 m, where the detection probability remains above 0.9, ensuring the highest possible detection efficiency while mitigating losses due to excessive path length and beam dispersion.

##### Detection efficiency vs UAV speeds.

**Fig 19:**
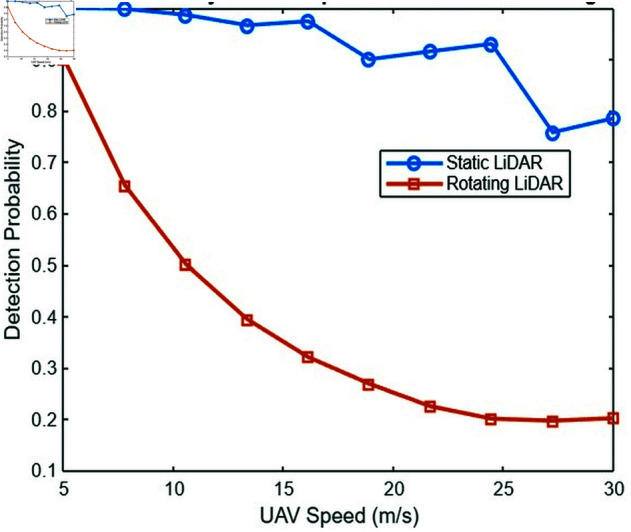
Impact of UAV speeds on the detection efficiency.

Fig 19 highlights the consistent advantage of static LiDAR over rotating LiDAR in UAV detection, particularly as UAV speed increases. Static LiDAR continuously monitors its FOV, ensuring a higher detection probability, while rotating LiDAR intermittently scans the environment, leading to potential blind spots between rotations. This fundamental difference explains why static LiDAR maintains a superior detection rate at all speeds tested.

At low UAV speeds (5–10 m/s), both systems perform well, with detection probabilities greater than 90% for static LiDAR and 80–90% for rotating LiDAR. However, as the speed of the UAV increases, the rotating LiDAR struggles to detect fast moving UAVs that may pass through its scanning gaps, causing the detection probability to drop below 50% at 30 m/s. In contrast, static LiDAR maintains a detection probability greater than 65% even at high speeds, confirming its robustness for real-time UAV tracking.

The trade-off comes in terms of energy consumption and hardware complexity. Although static LiDAR provides continuous coverage, it requires multiple sensors for wide-area monitoring, increasing cost and power demand. Rotating LiDAR, being a single moving unit, offers a more cost-effective and energy-efficient solution, but at the expense of lower detection reliability at high UAV speeds.

### 7.3 Recommended performance characteristics for accurate drone detection

Based on our simulation results, we propose key performance recommendations to optimize the detection capabilities of both LiDAR systems. These recommendations focus on maintaining a balance between detection accuracy, power efficiency, and real-time operation.

#### Static LiDAR system.

- Optimal Sensor Spacing: Detection efficiency is maximized with a sensor spacing of 0.02−0.03m, reducing coverage gaps while avoiding optical interference.
- Detection Range: The system achieves optimal detection at 50 m, where power attenuation remains manageable.
- Detection Frequency: The static system benefits from continuous data acquisition, ensuring real-time updates with minimal delay.
- Power and Sensitivity: A transmitted power of 5W per sensor and a detection threshold of 0.1mW ensure reliable UAV detection in various speed ranges.
- Coverage Geometry: A quasi-spherical sensor distribution ensures 360° monitoring, minimizing blind spots.

#### Rotating LiDAR system.

- Rotation Speed and Dwell Time: A balanced scan rate is necessary to maintain high detection accuracy while minimizing latency due to mechanical rotation.
- Detection Frequency Considerations: As the system relies on sequential scanning, optimizing the rotation speed is essential to avoid detection gaps.
- Detection Range: The system maintains optimal performance at 50 m, although adjustments are needed to counteract scanning delays.
- Power and Mechanical Efficiency: Although the static system requires fewer sensors than the static system, additional energy is needed for rotation. Efficient mechanical energy management is crucial to energy efficiency.

## 8 Conclusion and future directions

This study proposed and analyzed two LiDAR-based UAV detection systems: the multi-array static LiDAR and the one-array rotating LiDAR. These systems were developed to overcome the limitations of traditional detection methods such as radar, acoustic sensors, and RF detection, which often suffer from limited resolution, environmental interference, and challenges in distinguishing UAVs from background noise. Using laser-based detection, our approach delivers high spatial accuracy, continuous monitoring, and robust performance under various atmospheric conditions.

Our results indicate that the multi-array static LiDAR system provides instantaneous, high-resolution coverage, making it especially suitable for applications that demand real-time UAV detection with minimal scanning delays. However, this performance comes at the expense of higher power consumption and increased system complexity due to the need for multiple sensor arrays. In contrast, the one-array rotating LiDAR system is designed to be compact and energy efficient by employing a single sensor that rotates to cover a broad surveillance area. Although this approach reduces hardware costs and power consumption, it introduces detection delays and potential mechanical wear, which may limit its effectiveness in high-speed tracking scenarios.

Furthermore, our analysis confirms that the scanning frequency is a critical performance metric. The continuous operation of the static system produces a significantly higher effective detection frequency, which is beneficial in scenarios requiring immediate response. In contrast, the rotating system, because of its sequential scanning process, suffers from inherent limitations in its update rate, resulting in temporal gaps between observations. Although the rotating system can cover a larger area, it cannot match the near-instantaneous detection updates provided by the static configuration.

The quasi-spherical radar configuration used in both systems guarantees 360° angular coverage, thereby minimizing blind spots and enhancing detection reliability. Our findings also demonstrate that optimizing parameters such as beam divergence, inter-array spacing, and sensor placement is crucial for maximizing detection efficiency. The simulation results indicate that there is an optimal UAV observation range where the received power remains above the detection threshold while minimizing atmospheric losses. For static LiDAR, careful selection of interarray spacing is critical, as it must balance sensor overlap with coverage continuity. Similarly, for the rotating LiDAR, the rotation speed is a key factor: slower rotations improve detection accuracy by increasing dwell time, whereas faster rotations, while expanding coverage, lead to increased detection delays.

Future research should explore adaptive scanning techniques to dynamically adjust sensor focus based on UAV movement, thus improving real-time tracking. In addition, integrating machine learning algorithms could improve signal processing by filtering noise and accurately classifying UAVs. Further refinement of mathematical models to account for complex environmental factors such as moving obstacles, dynamic weather conditions, and multiple UAV targets will improve detection reliability. Finally, optimizing low-power LiDAR architectures and integrating complementary detection technologies (e.g., radar, acoustic sensors, and infrared imaging) could yield a versatile multi-modal UAV detection system suitable for diverse surveillance environments, including critical infrastructure protection, airport security, and military applications.
